# Precise prediction of launch speed for athletes in the aerials event of freestyle skiing based on deep transfer learning

**DOI:** 10.1038/s41598-023-31355-8

**Published:** 2023-03-15

**Authors:** Daqi Jiang, Hong Wang, Jichi Chen, Chuansheng Dong

**Affiliations:** 1 School of Mechanical Engineering and Automation, Northeastern University, Shenyang, China; 2 Key Research Center for Social Science, Shenyang Sport University, Shenyang, China

**Keywords:** Electrical and electronic engineering, Mechanical engineering

## Abstract

Automatically obtaining the launch speed are powerful guarantees for athletes in the aerials event of freestyle skiing to achieve good results. In most of the published studies describing athletes getting high scores, the assisting sliding distance depends entirely on the coach and even the athlete’s own experience, which may not be optimal. The main goal of the present paper is to use an acquisition system and develop an artificial neural network (ANN) model to automatically obtain the corresponding relationship between assisting sliding distance and speed. The influence of snow friction coefficient, wind speed, wind direction, slope, height and weight can be simulated in the Unity3D engine. The influence of temperature, humidity and tilt angle needs to be measured in real world by professional testers which is strenuous. The neural network is first trained by sufficient simulation data to obtain the encoded feature. Then, the information learned in simulation environment is transferred to another network. The second network uses the data taken from twenty professional testers. Compared with the model without transfer learning, the performance of proposed method has significant improvement. The mean squared error for the testing set is 0.692. It is observed that the speed predicted by the designed deep transfer learning (DTL) model is in good agreement with the experimental measurement results. The results indicate that the proposed transfer learning method is an efficient model to be used as a tool for predicting the assisting sliding distance and launch speed for athletes in the aerials event of freestyle skiing.

## Introduction

**Figure 1:**
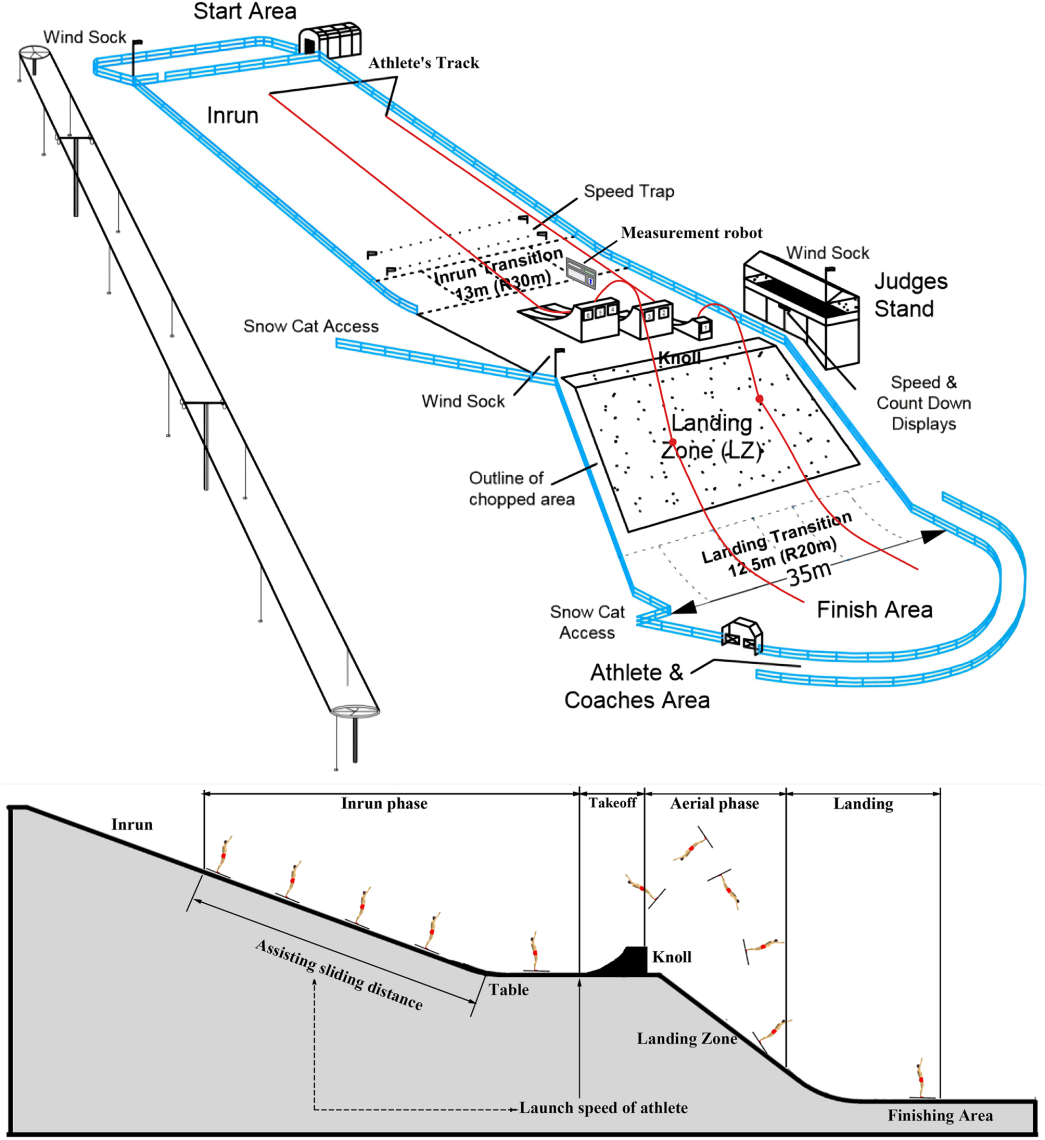
The diagram and side view of the aerial site.

The aerials event of freestyle skiing is a sport that attracts people’s attention around the world. The project mainly demonstrates the technique and flexibility of the athletes, and is very consistent with the athletes’ own sports characteristics and physical features^1^. Therefore, the aerials event of freestyle skiing has always been an important breakthrough for athletes to win gold in the Winter Olympics. The action composition of aerials event of freestyle skiing is mainly divided into four phases, namely the assisting sliding, take-off, the aerial and landing phase, respectively. Usually, these four phases are connected to each other, promote each other, and interact with each other. One of the key factors determining the success or failure of the action is the control of the height of the rise, that is, the control of the speed of the launch at the end of the assisting sliding phase. The diagram and side view of the aerial site is shown in Fig. 1 where the four phases and crucial launch speed point are presented in detail.

However, the speed of the launch is influenced by many factors. In the past competitions, the assisting sliding distance of the aerials event of freestyle skiing depends entirely on their coaches and even the athlete’s own experience, or the state of final determination is determined after repeated sliding tests, which may not be optimal. In addition, the conduction of outdoor trials is time-consuming and strenuous^2–4^. The assisting sliding speed is influenced by the snow conditions, environmental surroundings and sliding slope. The snow conditions include snow temperature, hardness, and texture^5–7^, all of which directly affect the friction coefficient of snow, which in turn affects the speed of sliding. The limitation of repeated sliding tests of athletes can be overcome by using a ski-snow tribometer system. A ski-snow tribometer system is preferred due to its objective, fast and reliable measurement. However, fewer works have been conducted on the development of the ski-snow tribometer system. The existing measurement systems can be classified into two categories: (1) linear tribometers^6,8^; (2) rotational tribometers^9^. A limitation of the existing systems is that they cannot meet the measurement of friction along the ski due to the size of samples and construction of systems. In addition, more importantly, besides coefficient of friction of the snow, the speed of athlete is also related to assisting sliding distance, wind speed, wind direction, slope, height, weight temperature, humidity and tilt angle. Unfortunately, the existing measurement model does not directly give the relationship between the speed and these factors to guide the athletes to achieve good results, which is unfavorable for the athletes.

In this paper, we proposed a novel algorithm to predict the speed of athlete and provide guidance on assisting sliding distance for athlete. The model comprehensively considers the effect of coefficient and captures the complex relationships between variables via studying the important features embedded in the data. Raw data was collected through test experiments to change the status quo that the assisting sliding distance entirely depends on the coach and even the athlete’s own experience.

ANNs are employed in various fields such as classification, pattern recognition, prediction, etc^10–12^. Recently, the applicability of ANN is increased by providing solution to engineering applications like groundwater monitoring, concrete strength prediction, hopper discharge rate prediction, and prediction of friction factor of pure water^12–15^. Naderpour et al.^16^ predicted the recycled aggregate concrete compressive strength with the help of ANN. The regression values of the selected network for training, validation and testing are 0.903, 0.89 and 0.829 respectively. An ANN is developed by Kumar et al.^15^ to predict the mass discharge rate from conical hoppers, and the chosen ANN model was capable of predicting the discharge rate of multi-component particle systems from different angle conical hoppers within ± 13% error. Cebi et al.^12^ developed an ANN model of friction factor in smooth and microfin tubes under heating, cooling and isothermal conditions. Results indicate that such system based on neural network could effectively predict the friction factor values of the flows regardless of their tube types. The main reason why ANNs are widely recognized is their effectiveness to settle complex engineering issues^17^. ANN abstracts the human brain neural network from the perspective of information processing, establishes a certain simple model, and forms different networks according to different connections. Therefore, ANN reveals the amazing ability of human brain modeling. To the best of our knowledge, Applications of ANN for predicting the assisting sliding distance for athletes in the aerials event of freestyle skiing are very rare in the literature. Up to date, there is no study regarding the prediction of assisting sliding distance. The research in this paper fills in the gaps in related fields and provides a basis for follow-up research.

In conference on neural information processing systems, researchers give a representative definition: transfer learning aims to transfer knowledge between similar but different domains, tasks and distributions. Later, with the further development of transfer learning, Pan and Yang^18^ gave a formal definition of transfer learning in 2010, which divides it into isomorphic transfer learning and heterogeneous transfer learning. In this paper, the problem to be solved is isomorphic transfer learning where the feature space and class label space of the source domain and target domain are the same but marginal distribution or conditional distribution is different. Bousmalis et al.^19^ proposed the domain separation network, whose network architecture consists of a common encoder, a source domain encoder, a target domain encoder, a common decoder, and a classifier, which separates domain-specific features while mining common domain features. Yan et al.^20^ proposed the weighted domain adaptation network, which designed a weighted maximum mean discrepancy based on category prior information to fit the source domain and the target domain. Li et al.^21^ proposed the joint adversarial domain adaptation method, which performs both feature-level and class-level confrontation. The former is used to reduce the marginal distribution between domains and the latter is used to reduce the conditional distribution. Transfer is now widely used in the learning of various knowledge, skills and social norms^22–24^.

## Results

The performance of designed DTL model for the prediction of the launch speed is tested in Baiqingzhai ski field whose freestyle skiing aerial skills venue meets international competition standards. The latitude and longitude of Baiqingzhai Ski Resort are 41.58093 and 123.7144 respectively. The experiment is conducted in four days with different weather to explore the influence of temperature and humidity. A total of 300 input–output data sets are generated based on the best 15 tests per professional tester. Out of the 300 input–output datasets, 240 datasets are used for training and the remaining 60 datasets are used for validation.

**Figure 2:**
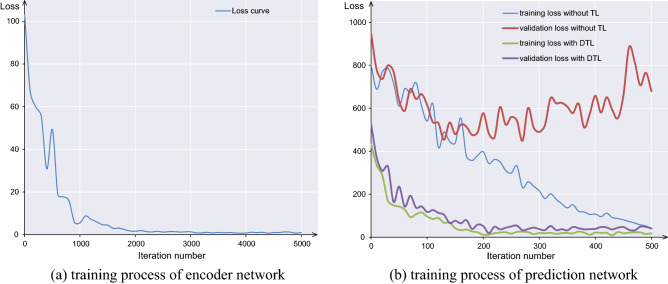
The loss curve during training process.

To train the neural network, the PC is equipped with 32.0 GB RAM, Core i7-9700F CPU 3.0 GHz, and the NVIDIA GeForce RTX 2060. The encoder network is first trained by 100,000 sets of simulation data with 100 batch size and 5000 epochs. The total time for training the encoder network is around 4 h. After training process, the parameters of hidden layers are frozen. The last hidden layer of encoder network and other three parameters in real world is the input of the prediction network. Label of data is the launch speed measured by equipment. The prediction network is trained by 240 sets of real-world data with 48 batch size and 500 epochs in around 20 min. The loss curve during training process is shown in Fig. 2. Apparently, the DTL model converges rapidly and the loss function of the training set and validation set is reduced synchronously. To demonstrate the effectiveness of the transfer learning, an experiment that the prediction network is directly trained by real-world data is conducted. The model without transfer learning converges very slowly. The loss function on the validation set increases in the later stage, and the phenomenon of overfitting soon appears.

The performance of the network trained is compared with only simulated data and only real-world data, and the network with transfer learning. The mean square error (MSE) are 4.280, 1.946 and 0.692, respectively. The result reveals that the proposed DTL model outperforms other models by a large gap. The performance of network with only simulated data is worst because of the domain shift between the simulation and real-world environments. The proposed DTL model integrates knowledge in the simulated environment to form encoded information and transfer it to the real environment. The MSE of the best neural network is 0.692. It is hence to conclude that the selected neural network is able to provide a good correlation between the environment parameters and the predicted launch speed. The MSE of network with only simulated data is worst because of the domain shift between simulation and real-world environment.

Since a method to determine the best neural network structure and optimal training method has not been developed, in the present work, the available datasets are trained, validated and tested by other network presented in^4,7^ until the network obtains the minimum MSE. According to number of input nodes, hidden layers, and hidden nodes, the two networks are respectively named Model 9-2-8 and Model 11-2-15. The training is automatically terminated after 5000 epochs. Table 1 lists different ANN structure tested during model fitting, in the light of training, validation and testing errors with change in the structure of neurons in hidden layer. Investigation of the Table 1 reveals that the proposed neural network outperforms other structure by a wide margin. It is hence to conclude that the selected neural network is able to provide a good correlation between the target and the predicted the launch speed for athletes in the aerials event of freestyle skiing.

**Table 1:** Performance of different ANN structure.

ANN structure	MSE		
	Learning set	Validation set	Testing set
Model 9-2-8	3.145	3.290	3.421
Model 11-2-15	2.517	2.991	3.145
Ours	**0.593**	**0.689**	**0.692**

Significant values are in [bold].

## Discussion

In this work, an acquisition system for coefficient of friction of the snow, wind speed, wind direction and tilt angle is developed and a novel DTL algorithm is proposed to predict the launch speed of athletes in the aerials event of freestyle skiing to change the status quo that the assisting sliding distance entirely depends on the coach and even the athlete’s own experience. The model comprehensively takes into account various factors that affect launch speed of athletes. Some of these factors can be simulated by the Unity3D engine, generating large amounts of data. For other parts, 20 athletes conduct experiments in real-world environments and obtain a small amount of data. Our proposed DTL algorithm effectively handles the quantitative imbalance and domain drift between the two sets of data. Through our experiments, we discovered that the DTL algorithm can learn useful information for simulation domain and transfer it to real-world domain and resulting in the best performance. The MSE of the neural network is 0.692 which significantly outperforms other models without transfer learning and meets the needs of athletes. The result shows that the proposed method can help coaches and athletes to choose an appropriate assisting sliding distance based on the environmental parameters at the time.

In future work, we will develop virtual reality systems based on existing neural network models. In this system, athletes can control the speed on different snow surfaces by adjusting the tile angle and attitude to improve the adaptability to different environments. The system can break the restrictions of the season on freestyle skiing.

## Methods

### Acquisition system

**Figure 3:**
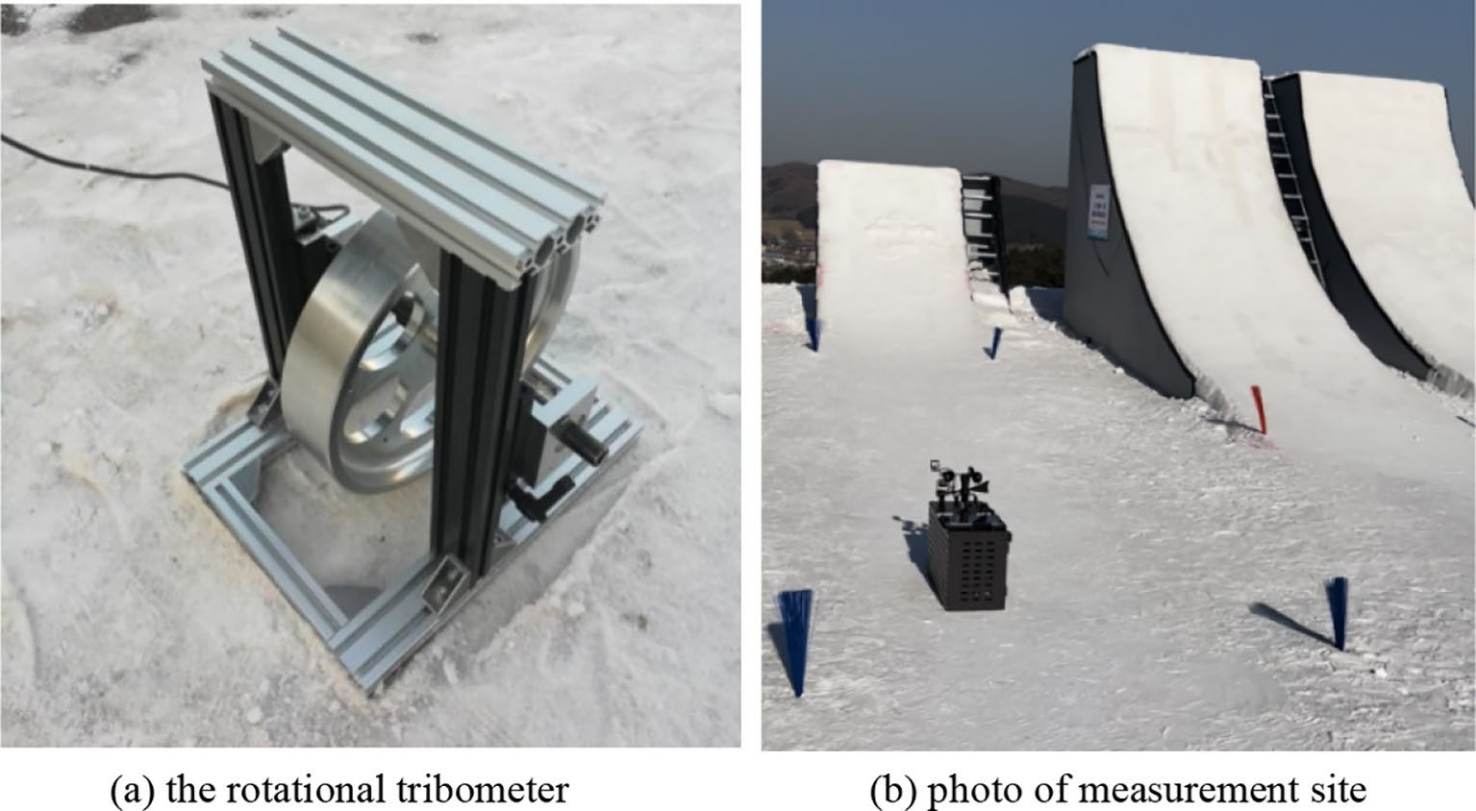
Overall structure of the measuring equipment.

The rotational tribometer is shown in Fig. 3a which consists of measuring rotation wheel subsystem, power starting subsystem, and supporting falling subsystem. More specifically, the measuring rotation wheel subsystem consists of rotation wheel, support shaft, ceramic bearing, bearing block, guiding rail, rail slider and rotational speed sensor. The power starting subsystem consists of stepper motor, electric slider, and separable transmission part. The supporting falling subsystem consists of frame, drop slider, and support frame. In the power starting subsystem, the stepper motor transmits power to the rotation wheel in the measuring rotation wheel subsystem through the separable transmission part, so that the rotation wheel reaches a certain speed. At this moment, the electric slider in the power starting subsystem drives the stepper motor out of the transmission. The electric slider in the supporting falling subsystem supports measuring rotation wheel subsystem to fall to the measured snow surface so that the outside surface of the rotation wheel is in contact with the measured snow surface, and the current rotating speed of the rotation wheel is recorded as *W*_1_ by the rotational speed sensor. When the rotation wheel is in contact with the measured snow surface for *T* seconds, the current rotating speed of the rotation wheel is recorded as *W*_2_, and the friction coefficient (*μ*) can be calculated by the following formula:

1 $$\begin{array}{c}\alpha =\frac{{W}_{1}-{W}_{2}}{T}\end{array}$$

2 $$\begin{array}{c}f=\frac{2J\left({W}_{1}-{W}_{2}\right)}{Td}\end{array}$$

3 $$\begin{array}{c}\mu =\frac{2J\left({W}_{1}-{W}_{2}\right)}{TNd}\end{array}$$

where *f*, *N*, *d*, *M* and *α* are the friction, the pressure, the diameter of rotation wheel, moment of inertia of the rotation wheel and deceleration of the rotation wheel, respectively.

**Figure 4:**
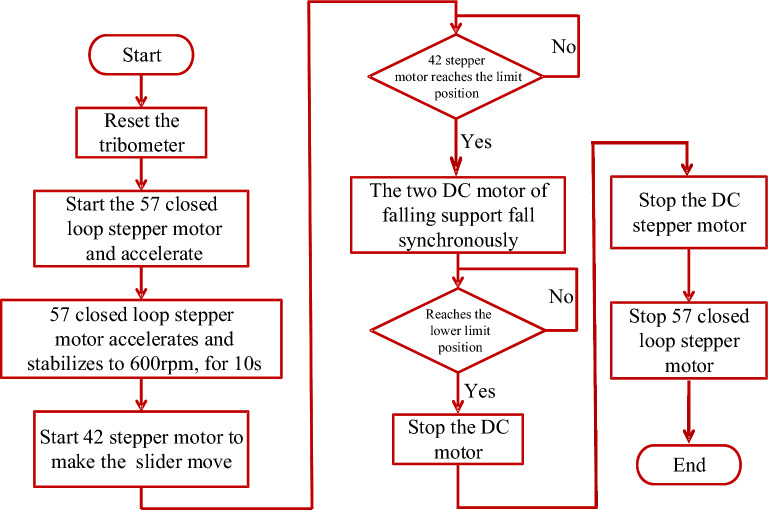
The flow chart of the control system.

According to the measurement process of the rotational tribometer, the design requirements of the control system are analyzed, and the stepper motor and the DC motor are controlled by the single chip microcomputer (SCM). Based on the analysis of the principle of SCM control, the asynchronous control of the stepper motor is realized, and the digital incremental PID control theory is used to realize the synchronous control of the two DC motors. In this research, the 57 closed loop stepper motor is driven by the HBS657 driver, the 42-stepper motor is driven by the DM320 driver, and the AQMH2407ND driver is used for the JGA25 geared motor. More specifically, the control flow of the four motors is as follows: press the start switch to reset the tribometer, then start 57 closed loop stepper motor and accelerate, 57 closed loop stepper motor accelerates and stabilizes to 600 rpm, for 10 s, and start the 42-stepper motor to make the slider move, the 42-stepper motor reaches the limit position, and the two motor of falling support fall synchronously until the lower limit position. The flow chart of the control system is shown in Fig. 4. Additionally, in order to get a more accurate prediction of the assisting sliding distance, wind speed, wind direction and tilt angle are also measured in this research. Since the angular velocity sensor requires high precision, a high-resolution photoelectric encoder (Omron E6H-CWZ3E) is used. The MCA420T-60-02 tilt angle sensor and SHT3X series temperature and humidity sensor module are selected, and the wind speed and wind direction sensor are RS-FSJT model and RS-FXJT model respectively. The photo of the entire measurement robot deployed in the ski resort is shown in the Fig. 3b.

### Participants

Twenty professional testers (aged from 20 to 30, mean = 23.75, SD = 2.99) participated in this study, which is approved by the ethics committee of The Northeastern University. Each participant performs 20 test runs and the best 15 tests per tester are selected for further analysis. The coefficients at the end of the assisting sliding stage are measured before each test. The assisting sliding distance and speed are also recorded.

### Data description and preprocessing

**Figure 5:**
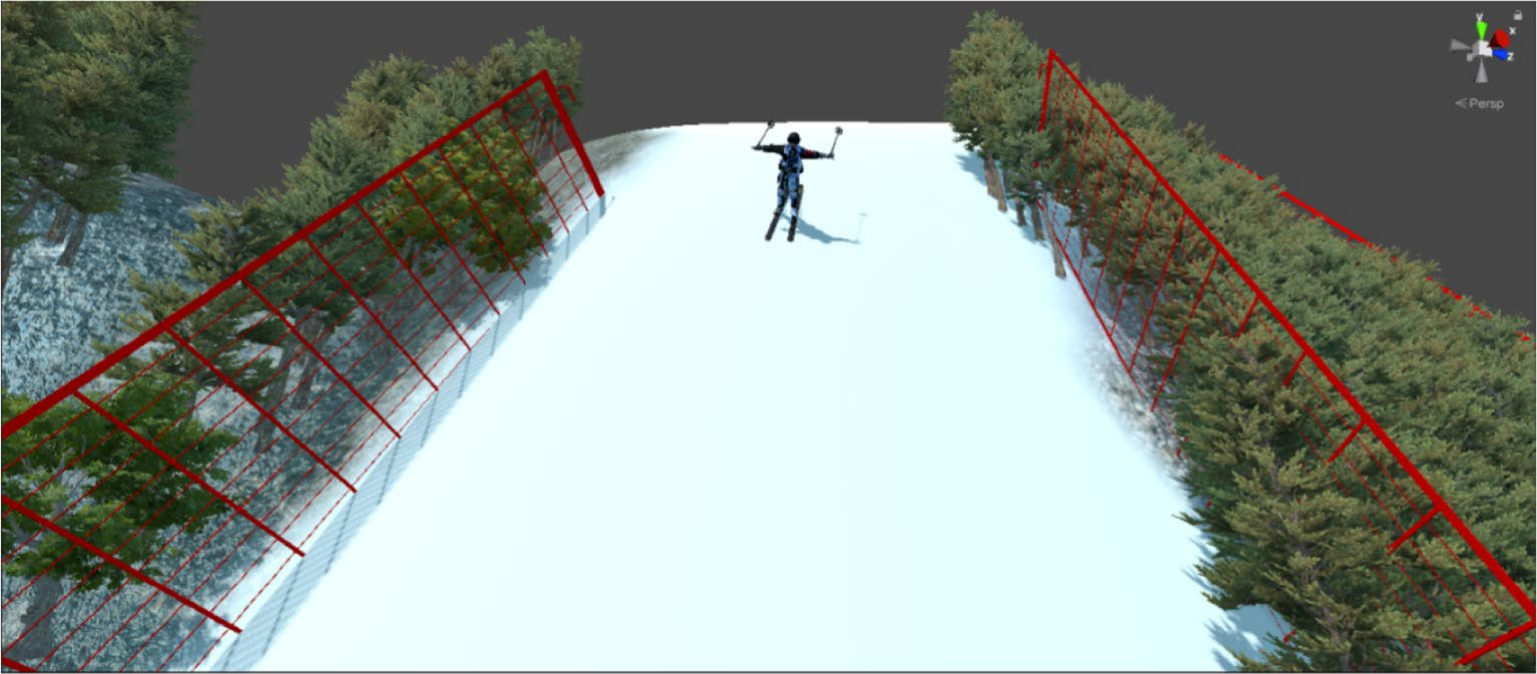
The virtual environment of the aerials event of freestyle skiing in Unity3D engine.

The dataset contains two parts: sufficient simulation data generated by Unity3D engine and fewer data in real world collected from recruited professional athletes. The first dataset includes snow friction coefficient, wind speed, wind direction, slope, assisting sliding distance, height and weight of athlete which can be simulated in the Unity3D engine. We set up a virtual environment of the aerials event of freestyle skiing which is shown in Fig. 5. A total of 100,000 sets of experiments were simulated. These parameters follow a Gaussian distribution and the mean and standard deviation of the parameters’ distribution are shown in Table 2. After five hours of calculations, all the data is easily completed. In addition to the parameters contained in the first dataset, the second dataset also includes temperature, humidity and tilt angle which are hard to be simulated. The data need to be measured in real world by professional testers which is time-consuming. Hence, it takes four days to collect a total of 300 sets of data. The wind speed and direction are converted into horizontal speed *v*_*x*_ and vertical speed *v*_*y*_. In addition, the data need to be normalized by the following equation to balance the diversity of parameter distribution.

4 $$ \begin{array}{*{20}c}    {\hat{y}^{{l\left( {i,j} \right)}}  = \frac{{y^{{l\left( {i,j} \right)}}  - \mu _{B} }}{{\sqrt {\left( {\sigma _{B}^{2}  + \epsilon } \right)} }}}  \\   \end{array}  $$

where ${y}^{l(i,j)}$ is raw value of data, $\hat{y}^{l(i,j)}$ is the normalized value of data, ${\mu }_{B}=\mathrm{E}\left[{y}^{l(i,j)}\right]$, ${\sigma }_{B}^{2}=\mathrm{Var}\left[{y}^{l(i,j)}\right]$, $\epsilon$ is a small constant added for numerical stability.

**Table 2:** The distribution of the parameters (add a correlation coefficient ρ = 0.9 between height and weight).

Physical quantity	Mean	Standard deviation
*v*_*x*_ (m/s)	6	2
*v*_*y*_ (m/s)	6	2
Snow friction coefficient	0.1	0.02
Slope (°)	23	2
Assisting sliding distance (m)	25	5
Height (m)	1.7	0.05
Weight (kg)	70	5

### Proposed deep transfer learning

**Figure 6:**
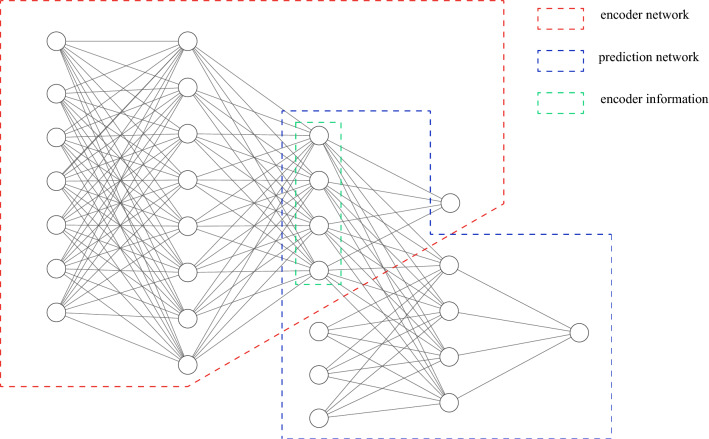
The structure of the proposed neural network.

As mentioned in the previous section, the data includes a large amount of simulation data as well as a small amount of real-world data. The algorithm needs to deal with the quantitative difference and domain shift of the two datasets. A novel supervised deep transfer learning method is proposed. The structure of the neural network is shown in Fig. 6. The red part called encoder network is first trained by simulation data with label. The inputs of the neural network are the seven parameters of the environment and the output is the prediction of the launch speed. The last layer of the hidden layer is deliberately designed to be narrow. In this layer, the most useful information is reserved and the previous layers are regarded as the encoder of the input. The blue part called prediction network is then trained by less real-world data. The inputs are the last hidden layer of encoder network and other three parameters. Note that the layers in encoder network include information learned in simulation data, so they are frozen in second training process.

The mean square error (MSE), which is the average squared difference between outputs and targets, is selected as the loss function. In addition, a L2 regularization is added to loss function of prediction network in order to prevent overfitting. *λ* is the penalty coefficient of the regularization item. The total loss function of prediction network is presented in the following equation:

5 $$ \begin{array}{*{20}c}    {L\left( \theta  \right) = \frac{1}{n}\sum\limits_{{i = 1}}^{n} {\left( {y_{i}  - \hat{y}_{i} } \right)^{2} }  + \lambda \sum\limits_{{i = 1}}^{m} {\sum\limits_{{k = 1}}^{s} {W_{{ik}}^{2} } } }  \\   \end{array}  $$

The ANN models are trained using Adam optimization algorithm. The activation function is ReLU. The workflow of the DTL model is shown in Algorithm 1. Finally, the data of simulation and twenty participants are randomly divided into training and test sets and 80% of the data for training of the model and 20% for validation.
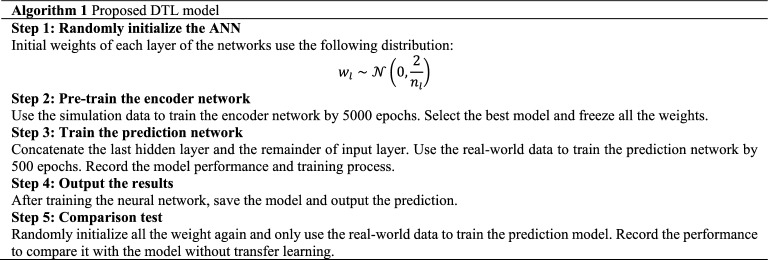


## Data Availability

The datasets generated during and/or analysed during the current study are not publicly available due to the confidentiality agreement but are available from the corresponding author on reasonable request.
