# Author Correction: Precise prediction of launch speed for athletes in the aerials event of freestyle skiing based on deep transfer learning

**DOI:** 10.1038/s41598-023-34472-6

**Published:** 2023-05-08

**Authors:** Daqi Jiang, Hong Wang, Jichi Chen, Chuansheng Dong

**Affiliations:** 1 School of Mechanical Engineering and Automation, Northeastern University, Shenyang, China; 2 Key Research Center for Social Science, Shenyang Sport University, Shenyang, China

Correction to: *Scientific Reports* 10.1038/s41598-023-31355-8, published online 15 March 2023

The original version of this Article contained errors in the Author contributions and Acknowledgements sections.

The Author Contributions section,

“D.J.: formal analysis, methodology, software, data collection, validation, visualization, conceptualization, writing—original draft, writing—reviewing and editing. H.W.: investigation, funding acquisition, conceptualization, resources, project administration and supervision. J.C.: data curation. C.D.: visualization.”

now reads:

“D.J.: formal analysis, methodology, software, data collection, validation, visualization, conceptualization, writing—original draft, writing—reviewing and editing. H.W.: investigation, conceptualization, resources, project administration and supervision. J.C.: data curation. C.D.: visualization and funding acquisition.”

In addition, the digit of the Project Code in the Acknowledgements section was incorrect.

“The authors received the support from The National Key R&D Program of China (2021YFF0306405). The authors appreciate the funding organization for their financial supports. The authors would also like to thank the Baiqingzhai ski resort in Shenyang of China for its full support.”

now reads:

“The authors received the support from The National Key R&D Program of China (2021YFF0306400). The authors appreciate the funding organization for their financial supports. The authors would also like to thank the Baiqingzhai ski resort in Shenyang of China for its full support.”

The original Article has been corrected.

